# Depression and dementia prevention: A scoping review of non‐pharmacological interventions for the treatment of depression in mid‐to‐older age minority ethnic populations

**DOI:** 10.1002/alz70860_097315

**Published:** 2025-12-23

**Authors:** Amirah Akhtar, Emmanuel Sunday Nwofe, Sahdia Parveen, Karen Windle

**Affiliations:** ^1^ University of Bradford, Bradford, West Yorkshire, United Kingdom

## Abstract

**Background:**

Depression is a potentially modifiable risk factor for dementia. People from minority ethnic (ME) backgrounds are at greater risk of developing depression but are less likely to receive treatment, perpetuated by health inequalities. Non‐pharmacological interventions are recommended as the first‐line treatment for depression and may potentially play a preventative role for longer term conditions such as dementia. This scoping review aimed to address a critical gap by mapping international evidence on non‐pharmacological interventions specifically targeting depression in ME populations aged 40 and above, an area overlooked in traditional reviews.

**Method:**

A scoping review was performed up to December 2024 using Medline, PsycINFO, CINAHL, Psychology and Behavioral Sciences Collection, Allied and Complimentary Medicine Database and Embase. Articles reporting on non‐pharmacological interventions targeting depression in the ME population, with a mean age of 40 and above were included. Two authors independently screened the articles, following the Joanna Briggs Institute guidelines.

**Result:**

Twenty‐one studies were included. Six interventions were identified: Cognitive Behaviour Therapy, Behavioural Activation, Mindfulness‐based, Logo‐Autobiography, Social Group intervention and Reminiscence Therapy. Interventions were adapted for ME populations via translation of materials and delivering the intervention in the target language. Interventions were also adapted to the ‘local context’ such as incorporating cultural norms and understanding of depression. Most of the studies were conducted in the USA with African American populations. This review found a lack of non‐pharmacological interventions for depression in people of ME backgrounds in the UK.

**Conclusion:**

A need for ME focused non‐pharmacological interventions for depression in the UK was indicated. A greater focus is required on recruiting people of ME backgrounds over the age of 40 for randomised controlled trials. This is an under‐represented group both in research and within mental health services. It is recommended that non‐pharmacological interventions for depression are culturally adapted and co‐produced with the target population. Cognitive deficits in depression are common but overlooked, it is recommended that assessment of cognition is made part of treatment. This will identify areas of need to support functional recovery of depression, to reduce the risk of relapse, and potentially prevent long‐term conditions such as dementia.

